# Dynamic Ultrasound of the Infrapatellar Fat Pad for Detecting Anterior Cruciate Ligament Deficiency: A Biomechanical Cadaveric Proof-of-Concept Study

**DOI:** 10.3390/diagnostics16071097

**Published:** 2026-04-05

**Authors:** Yoshiyuki Tokuda, Tsuneo Nakamura, Yoshitake Shiraishi, Kiyomi Hori, Hiroaki Okuda, Noriyuki Ozaki

**Affiliations:** 1 Department of Functional Anatomy, Graduate School of Medical Science, Kanazawa University, Kanazawa 9200934, Japan; 2 Department of Functional Morphology, Faculty of Pharmacology, Osaka Medical and Pharmaceutical University, Osaka 5691094, Japan

**Keywords:** infrapatellar fat pad, anterior cruciate ligament, ultrasound, adipo-follicular segment, infrapatellar plica

## Abstract

**Background/Objectives**: Diagnosing anterior cruciate ligament (ACL) insufficiency, particularly partial tears, remains challenging with standard static imaging. This study introduces a novel conceptual approach: assessing the dynamic kinematics of the infrapatellar fat pad (IPFP) as an indirect marker of ACL deficiency. **Methods**: In this biomechanical proof-of-concept study, dynamic ultrasound tracked IPFP kinematics in eight cadaveric knees evaluated in intact (Control), Sham, and Torn ACL states during passive flexion. The primary endpoints were (i) the absolute anteroposterior position at 90° (*y*_2_ − *y*_1_) and (ii) the posterior displacement during the 60–90° flexion arc (ΔY 60–90°). **Results**: ACL deficiency significantly altered deep-flexion IPFP kinematics. For ΔY 60–90°, the Torn ACL group demonstrated a substantial loss of posterior excursion compared to the Control group (Mean ± SD: −0.25 ± 1.03 vs. 2.88 ± 1.29 mm; Welch’s *p* < 0.001; Hedges’ g = 2.54, 95% CI: 1.18 to 3.89) and the Sham group (3.46 ± 1.63 mm; *p* < 0.001; g = −2.57, 95% CI: −3.90 to −1.25). Consequently, for *y*_2_ − *y*_1_ at 90°, the Torn ACL group remained abnormally anterior versus Control (*p* = 0.003; g = −1.97) and Sham (*p* < 0.001; g = −1.82). **Conclusions**: ACL deficiency induces a distinct reduction in posterior IPFP displacement. While these massive effect sizes establish a strong biomechanical rationale, this study serves as a foundational proof-of-concept. Large-scale in vivo clinical trials are strictly required to validate its diagnostic utility before clinical implementation.

## 1. Introduction

Diagnosing anterior cruciate ligament (ACL) injuries, particularly partial tears and subtle functional instability, remains a significant clinical challenge [1,2,3,4]. While magnetic resonance imaging (MRI) is the recognized gold standard [5], its static nature often fails to capture the dynamic incompetence of the ligament [6]. Although dynamic ultrasonography has emerged as a valuable point-of-care alternative, existing ultrasound metrics—such as anterior tibial translation or morphological signs [7,8,9,10,11]—predominantly quantify gross osseous laxity or ligamentous continuity. Consequently, there remains a critical diagnostic gap: current imaging strategies fail to reliably capture the fine, intra-articular soft-tissue kinematics that may serve as early indicators of functional deficiency.

To address this gap, we focused on the dynamic behavior of the infrapatellar fat pad (IPFP). The IPFP is an intracapsular structure mechanically and anatomically linked to the ACL and the anterior compartment [12,13,14,15,16]. In a healthy knee, ACL tension guides normal femoral–tibial kinematics during flexion. When the ACL is compromised, the resulting altered kinematics (e.g., femoral posterior subluxation) distort the compressive forces within the joint. We propose that the IPFP—specifically its highly compliant adipo-follicular segment (AFC-SEG)—acts as a sensitive biomechanical sensor, where its sagittal plane mobility is directly restricted by these altered intra-articular compressive forces. Thus, the AFC-SEG provides a promising window into these otherwise occult biomechanical dynamics.

Therefore, we hypothesized that the dynamic ultrasonographic assessment of IPFP kinematics (specifically the reduction in AFC-SEG mobility during deep flexion) could serve as a novel, indirect biomechanical marker for ACL deficiency. To test this hypothesis, we designed a biomechanical proof-of-concept study utilizing Thiel-embalmed cadavers, which preserve near-physiological soft-tissue flexibility. By quantitatively tracking the dynamic displacement of the AFC-SEG under controlled intact, sham, and ACL-deficient conditions, this study aims to verify the kinematic alterations induced by ACL tears and establish a foundational rationale for utilizing IPFP dynamics as a complementary diagnostic tool.

## 2. Materials and Methods

### 2.1. Identification of the Target Region (Macroscopic and Histological Assessment)

**Figure 1:**
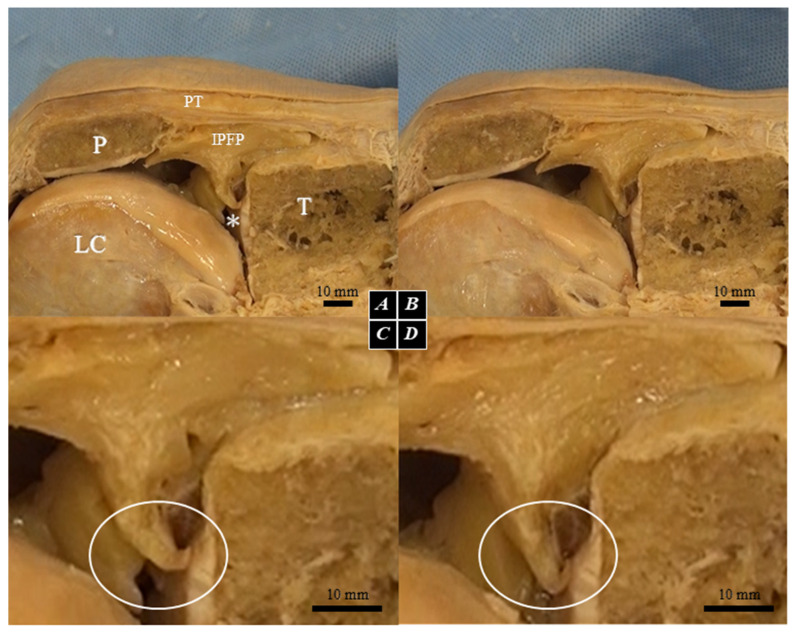
Gross anatomy of the IPFP embalmed in formalin. Lateral view at 90° knee flexion. (**A**) specimen with ruptured ACL; (**B**) specimen with intact ACL; (**C**) expanded (**A**); (**D**) expanded (**B**); P: patella; LC: lateral condyle of femur; PT: patellar tendon; T: tibia; *: lateral meniscus; white circle: morphologically changed region in the IPFP.

**Figure 2:**
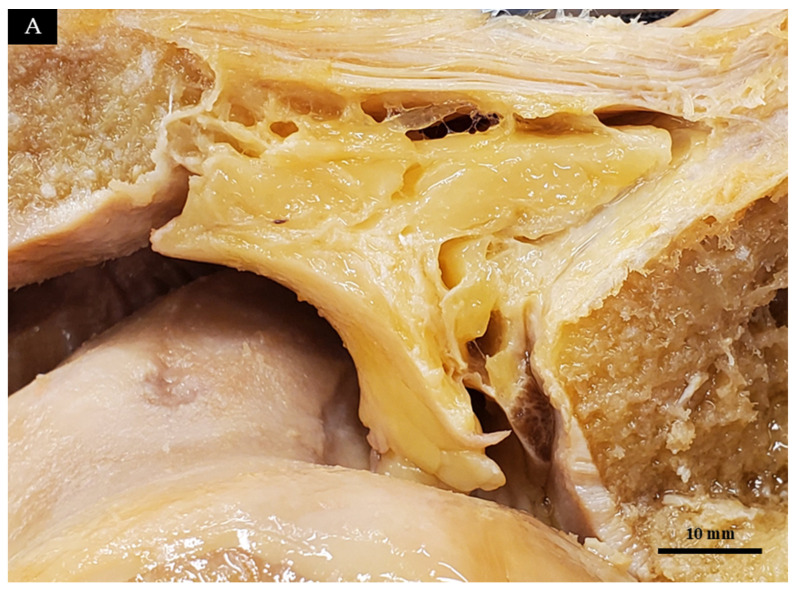

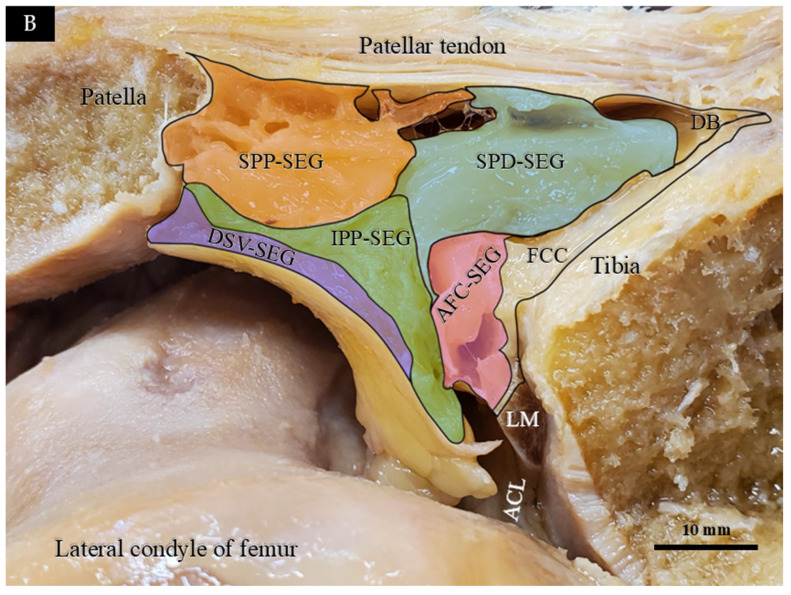
Segmental division of a formalin-embalmed IPFP. Lateral view at 30° of knee flexion. (**A**) Macroscopic view of the IPFP. (**B**) Color-coded representation of the segmental divisions. DB: deep infrapatellar bursa; FCC: fibro-capsular complex; LM: lateral meniscus.

**Figure 3:**
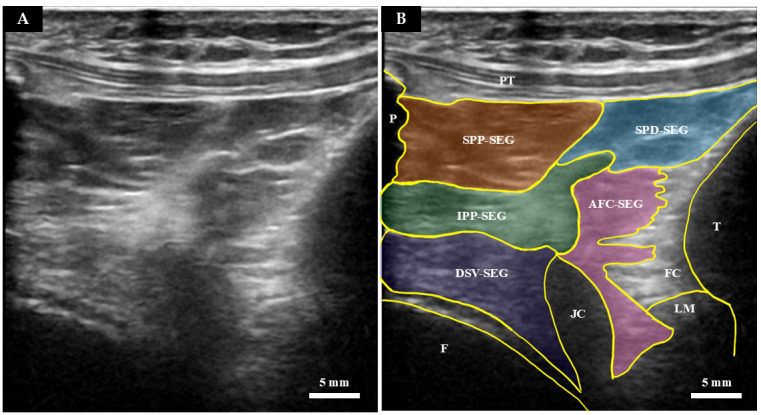
Long-axis ultrasound image of a Thiel-embalmed IPFP. (**A**) Original ultrasound image. (**B**) Color-coded representation of the segmental divisions. P: patella; PT: patellar tendon; T: tibia; F: femur; JC: joint cavity; LM: lateral meniscus; FC: fibro-capsular complex.

To determine the optimal region of the IPFP for dynamic tracking, we initially evaluated formalin-embalmed knees. Macroscopic observation across a 0° to 90° flexion range revealed that the most significant morphological disparity between the intact and ACL-deficient states occurred at 90° of flexion (Figure 1). Subsequently, the IPFP was macroscopically subdivided into five anatomical segments (Figure 2 and Figure 3, Table 1). We identified the adipo-follicular segment (AFC-SEG)—which specifically contains vesicle-like spaces into which the joint cavity extends—as the highly compliant region corresponding to the morphological changes observed in Figure 1. Histological analysis (H&E staining) confirmed the connective tissue and adipocyte architecture of this segment. Because these anatomical features corresponded with the region of maximal dynamic change observed in our preliminary clinical ultrasounds, we exclusively designated the AFC-SEG as our region of interest for kinematic tracking.

### 2.2. Thiel-Embalmed Specimens and Demographic Justification

To evaluate dynamic soft-tissue kinematics, we utilized Thiel-embalmed specimens, which uniquely retain near-physiological flexibility and water content. The study included 8 knees from 5 cadavers (5 females; mean age 93.4 years; range 83–100 years). Knees with pre-existing ACL tears or severe osseous deformities were excluded. We acknowledge that the extremely advanced age of the donors contrasts with the typical younger, athletic demographic affected by acute ACL tears. However, given the necessity of utilizing a model that permits highly realistic dynamic joint manipulation, Thiel-embalmed cadavers remain the most suitable ex vivo platform for this initial proof-of-concept. Furthermore, due to the highly limited availability of these specialized specimens, an a priori sample size calculation was unfeasible; the sample size was inherently constrained by the maximum number of viable specimens accessible.

### 2.3. Ultrasound-Guided Marking and Potential Biases

**Figure 4:**
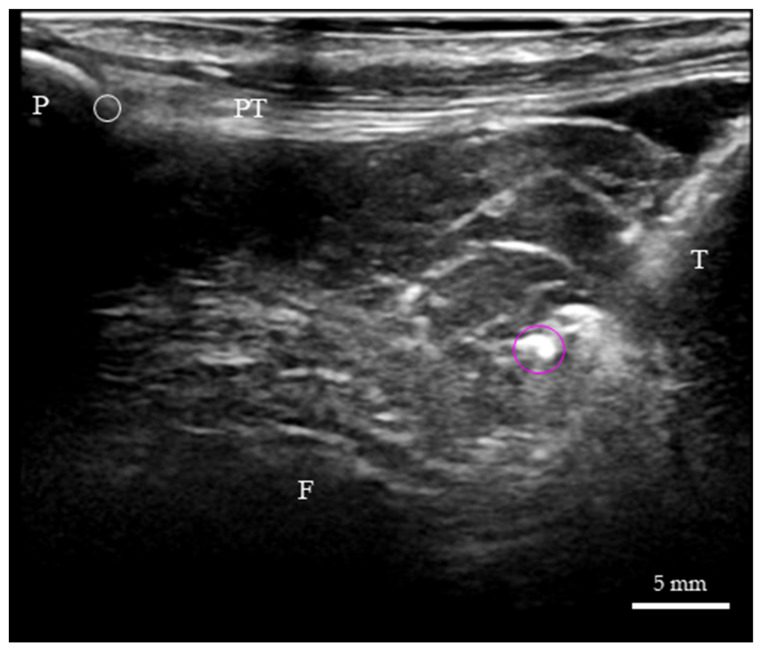
Long-axis ultrasound image of a Thiel-embalmed IPFP. A 14G needle is inserted into the AFC-SEG using an out-of-plane technique. white circle: anchor point; purple circle: 14G needle; P: patella; PT: patellar tendon; T: tibia; F: femur.

A linear ultrasound transducer (4–13 MHz, FUJIFILM Corp., Tokyo, Japan) was applied to the anterior knee with standardized B-mode settings optimized for superficial soft tissue. Under out-of-plane ultrasound guidance, a 14G needle with a cannula (Surf-lok 14G 2 1/2″, TERUMO Corp., Tokyo, Japan)was inserted into the AFC-SEG. A metallic marker (clip, ~10 mm) was deployed, and the needle and cannula were subsequently withdrawn (Figure 4). We recognize that the 14 G needle tract and the metallic clip itself may theoretically induce local tissue drag or micro-structural alterations. However, this marking technique was strictly necessary to establish a highly visible, reliable internal anchor for precise kinematic tracking. Any resulting mechanical bias was considered minimal and acted systematically across all tested conditions, thereby preserving the validity of relative comparisons.

### 2.4. Passive Flexion Protocol and Surgical Interventions

**Figure 5:**
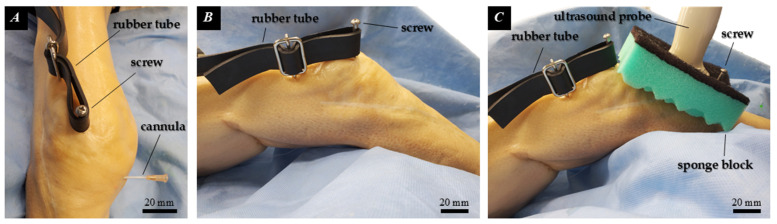
A Thiel-embalmed right knee at 45 knee flexion. (**A**) Anterior view; (**B**) Lateral view; (**C**) Lateral view demonstrating ultrasound probe application.

The ultrasound probe was secured to the anterior knee using a sponge block, with the imaging plane continuously verified against anatomical landmarks (inferior patellar pole and tibial contour). To simulate patellar tracking, a percutaneous screw in the patella was connected to a rubber tube, providing constant proximal tension (Figure 5). The knee was subjected to gravity-based passive flexion, and clip positions were recorded at 0°, 30°, 60°, and 90° (Control group). While this passive protocol fundamentally lacks active physiological loading—particularly the quadriceps tension known to influence IPFP mechanics in vivo—it was intentionally selected to ensure high reproducibility and isolated assessment of ligamentous influence across sequential states. Following the intact assessment, a posterior approach through the popliteal fossa was utilized to expose the ACL origin without disrupting the anterior capsular or extensor mechanics. The joint was re-sutured without transecting the ACL, and measurements were repeated (Sham group). Finally, the popliteal incision was reopened, the ACL was completely transected at its femoral origin, re-sutured, and final measurements were recorded (Torn ACL group).

### 2.5. Quantitative Kinematic Assessment

To eliminate observation bias, all ultrasound images were anonymized, blinding the ImageJ operator (version 1.53t; National Institutes of Health, Bethesda, MD, USA; nih.gov) to group assignments and flexion angles. The patellar anchor point (*x*_1_, *y*_1_) and the metallic clip (*x*_2_, *y*_2_) were plotted. Pixel measurements were converted to absolute millimeters (mm) using a standardized spatial calibration factor (0.0838 mm/pixel). Absolute coordinates (*x*_2_ − *x*_1_, *y*_2_ − *y*_1_) and interval displacements (ΔX, ΔY) were calculated for the 0–30°, 30–60°, and 60–90° flexion arcs. A positive Y-value indicated anterior displacement relative to the patellar anchor point (*x*_1_, *y*_1_). Five independent measurements per knee were performed by a single examiner to assess intra-rater reliability.

### 2.6. Statistical Analysis

Statistical analyses were performed using SPSS ver. 27.0 (IBM Corp., Armonk, NY, USA). To strictly account for specimen non-independence (8 knees from 5 donors), a Linear Mixed-Effects Model (LMM) with donor ID as a random intercept was employed for primary endpoints. Paired *t*-tests verified that the Sham procedure did not significantly alter kinematics compared to the Control. Intra-rater reliability was assessed via the intraclass correlation coefficient (ICC) based on a two-way mixed-effects model [17]. For three-group comparisons, Welch’s ANOVA and Welch’s *t*-tests were utilized due to unequal variances, with Bonferroni adjustments. To rigorously address the small sample size, standardized effect sizes were calculated using Hedges’ g with 95% confidence intervals. Exact permutation tests verified *p*-value robustness, and a post hoc power analysis was conducted. Significance was set at *p* < 0.05.

### 2.7. Ethics Statement

The study protocol was approved by the Institutional Ethics Committee of Kanazawa University (Approval No. 2882, Date: 17 October 2018). Written informed consent for the research use of cadaveric tissues was obtained from the donors’ families according to institutional policy. Because no living subjects were involved, standard clinical patient informed consent did not apply. All procedures were conducted in strict compliance with institutional and national ethical guidelines for cadaveric research.

## 3. Results

### 3.1. Histological Findings

**Figure 6:**
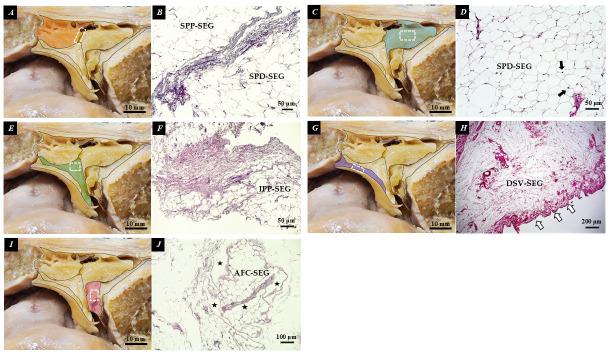
H&E staining of individual SEGs. (**A**) color-coded SPP-SEG; (**B**) stained between Ia and SPD-SEG; (**C**) color-coded SPD-SEG; (**D**) stained SPD-SEG; (**E**) color-coded IPP-SEG; (**F**) stained IPP-SEG; (**G**) color-coded DSV-SEG; (**H**) stained DSV-SEG; (**I**) color-coded AFC-SEG; (**J**) stained AFC-SEG; white dotted frame: tissue section sampling area; black arrow: nucleus; white arrow: synovial cells; black star: follicular region.

Histological analysis using H&E staining revealed distinct characteristics for each SEG of the IPFP. In the SPP-SEG and SPD-SEG, adipocytes were densely packed, and ubiquitous nuclei were observed. Connective tissue was clearly visible between the SPP-SEG and SPD-SEG. The IPP-SEG was predominantly composed of connective tissue. Synovial cells were identified in the DSV-SEG. Notably, the AFC-SEG contained spaces resembling follicular regions, distinct from the other SEGs (Figure 6).

### 3.2. Qualitative Ultrasound Observations

Qualitatively, along the *X*-axis (proximal–distal direction), the displacement of the metallic markers at 60° and 90° of flexion appeared greater in the Torn ACL and Sham groups than in the Control group. Along the *Y*-axis (anterior–posterior direction), while displacement magnitudes fluctuated slightly at early flexion angles (30° and 60°), a distinct and consistent pattern emerged at 90° of flexion. Specifically, the Torn ACL group exhibited a marked anterior positioning of the marker (indicated by higher Y-values) compared to both the Control and Sham groups.

### 3.3. Quantitative Kinematic Assessment and Reliability

**Figure 7:**
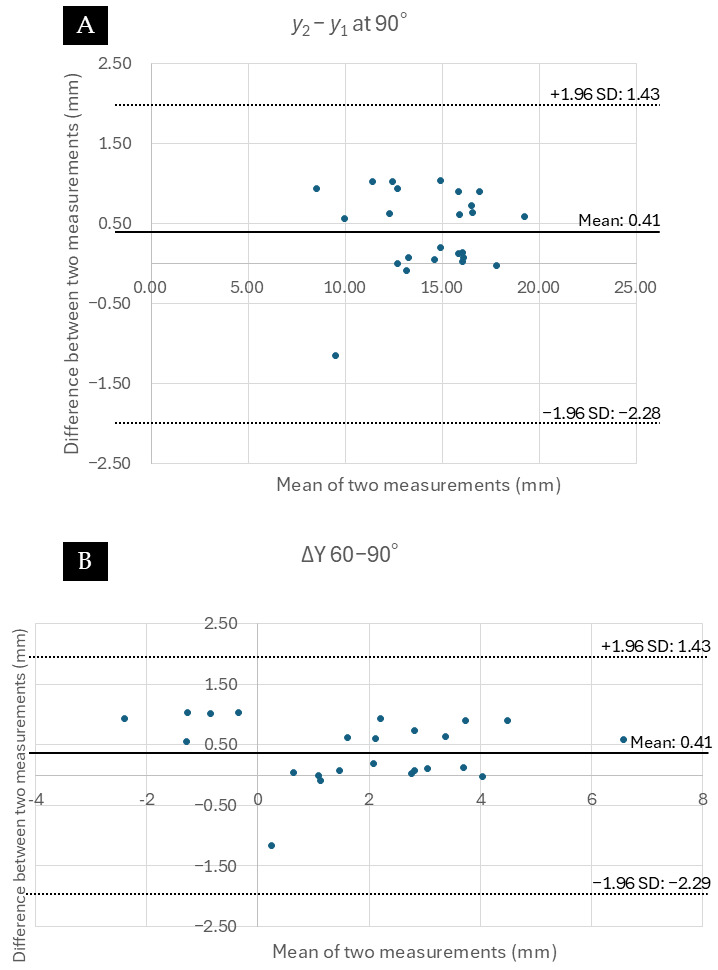
Bland–Altman plots assessing intra-rater reliability. The plots display the agreement between two repeated measurements performed by a single examiner. The *x*-axis represents the mean of the two measurements (mm), and the *y*-axis represents the difference between the two measurements (mm). (**A**) Analysis of the absolute anterior–posterior position (*y*_2_ − *y*_1_) at 90° of flexion. (**B**) Analysis of the posterior displacement during the 60–90° flexion arc (ΔY 60–90°). In both plots, the differences are distributed close to zero without discernible systematic bias, supporting the reproducibility of the ultrasound assessment method.

**Figure 8:**
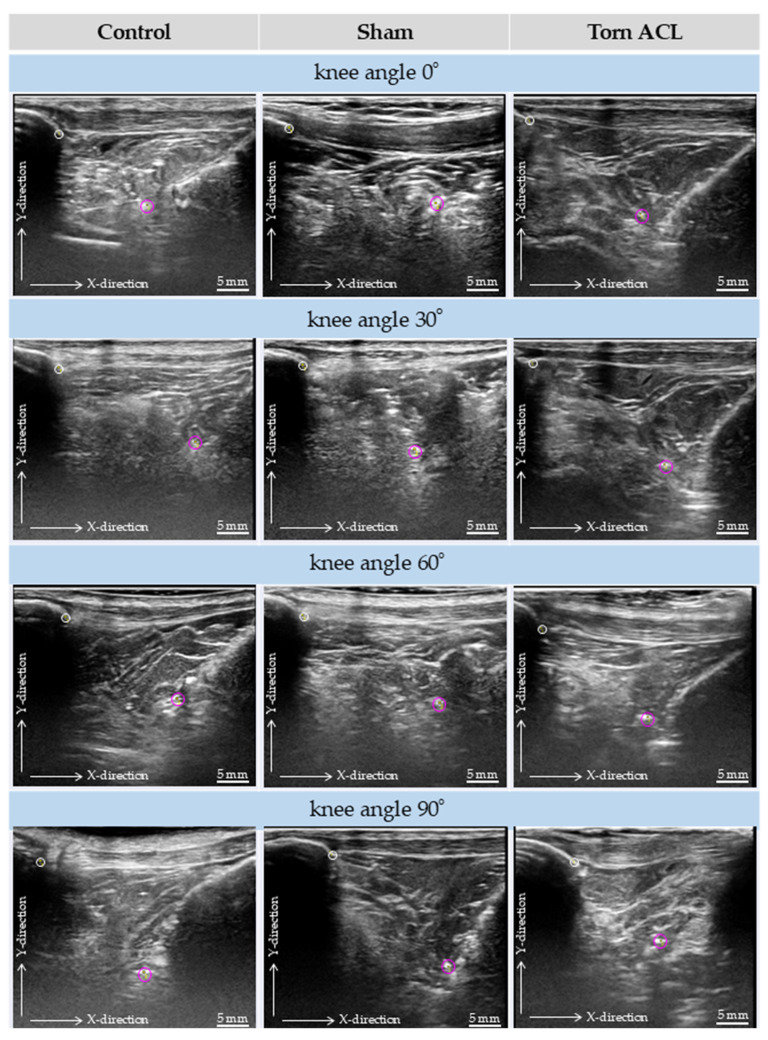
Long-axis ultrasound images of the IPFP in the three groups, visualized at the four set flexion angles. The metallic clip (purple circle) is inserted into the AFC-SEG, and the anchoring point is indicated by the white circle.

The intra-rater reliability for reproducing the exact ultrasound plane (quantified by the distance from the inferior pole of the patella to the metal clip) was excellent (single-measure ICC = 0.954, 95% CI: 0.881–0.989, *p* < 0.001). Bland–Altman plots confirmed high agreement between repeated measurements, showing a random distribution of differences around zero with no evidence of fixed or proportional bias (Figure 7). Reliability for the coordinate placements was also excellent (ICC > 0.953). To maintain narrative focus, the complete datasets of absolute coordinates (*x*_2_ − *x*_1_, *y*_2_ − *y*_1_) and interval displacements at all flexion angles are provided in Supplementary Tables S1 and S2. While *X*-axis displacement showed no significant inter-group differences, the absolute anterior–posterior position at 90° (*y*_2_ − *y*_1_) and the posterior displacement during the 60–90° arc (ΔY 60–90°) were conspicuously altered in the Torn ACL group (Figure 8).

### 3.4. Primary Endpoints and Statistical Analysis

Data normality was confirmed for the primary endpoints (*y*_2_ − *y*_1_ at 90° and ΔY 60–90°) using the Shapiro–Wilk test (*p* > 0.05). Initial comparisons revealed no significant differences between the Control and Sham groups for any coordinate parameters at any flexion angle (all *p* > 0.05). Specifically, at 90° of flexion, the mean difference in the anterior–posterior position (*y*_2_ − *y*_1_) between Control and Sham was minimal (+0.56 mm; *p* = 0.36), and no significant difference was observed in the proximal–distal position (*x*_2_ − *x*_1_; *p* = 0.76).

The most prominent finding of this study emerged during deep flexion. For the primary endpoint ΔY 60–90°, the Torn ACL group demonstrated a substantial loss of posterior excursion compared to the Control group (Mean ± SD: −0.25 ± 1.03 mm vs. 2.88 ± 1.29 mm; Welch’s *p* < 0.001; Hedges’ g = 2.54, 95% CI: 1.18 to 3.89). A comparably significant reduction was found against the Sham group (3.46 ± 1.63 mm; *p* < 0.001; Hedges’ g = −2.57, 95% CI: −3.90 to −1.25).

**Figure 9:**
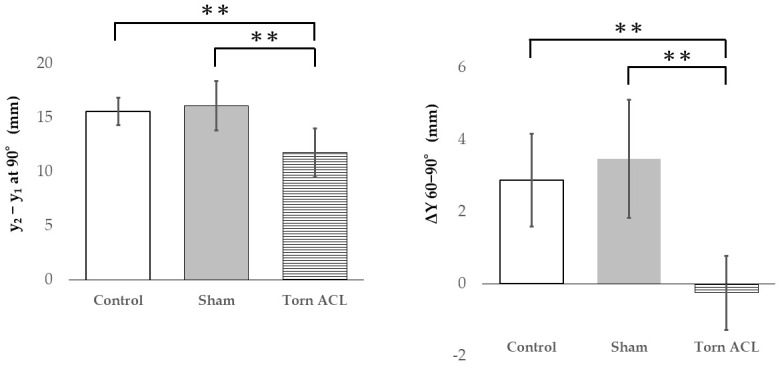
*Y*-axis coordinate (*y*_2_ − *y*_1_) at 90° flexion and *Y*-axis displacement between 60° and 90° flexion. Data are shown as mean ± SD, ** *p* < 0.01.

Consequently, for the absolute position at 90° (*y*_2_ − *y*_1_), the Torn ACL group remained significantly more anterior compared to both the Control group (Welch’s *p* = 0.003; Hedges’ g = −1.97, 95% CI: −3.16 to −0.78) and the Sham group (Welch’s *p* = 0.0008; Hedges’ g = −1.82, 95% CI: −2.98 to −0.66) (Figure 9). Exact permutation tests confirmed the robustness of these differences (*p* < 0.01). A post hoc power analysis verified that these massive effect sizes (Hedges’ g > 1.8) provided sufficient statistical power. All non-parametric datasets analyzed (e.g., *X*-axis intervals) showed no significant inter-group differences (*p* > 0.05).

### 3.5. Macroscopic Verification of the Marker Position

**Figure 10:**
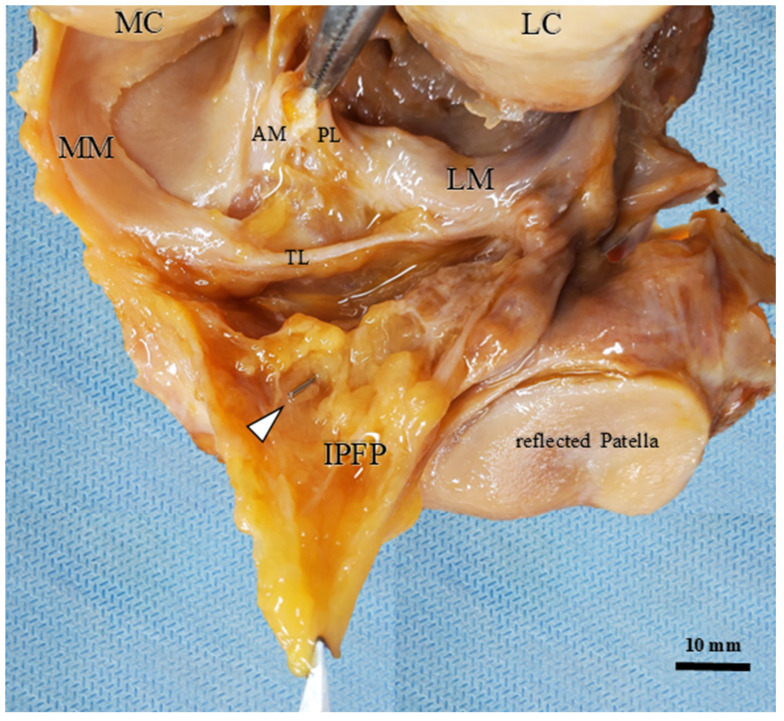
Macroscopic verification of the metallic clip position. The anterior dissection confirms that the clip (white arrowhead) is located within the AFC-SEG. The anteromedial (AM) and posterolateral (PL) bundles of the ACL are torn. The IPFP is held with tweezers. AM: anteromedial bundle of ACL; PL: posterolateral bundle of ACL; MC: medial condyle; LC: lateral condyle; MM: medial meniscus; LM: lateral meniscus; TL: transverse ligament.

Following the completion of all dynamic ultrasonographic assessments, an anterior dissection was performed to macroscopically verify the final position of the marker. In all specimens, it was visually confirmed that the metallic clip was accurately located within the target AFC-SEG. Specifically, the clips were found positioned between the IPP and the anterior horns of the menisci, with no evidence of migration into adjacent segments or joint impingement during the flexion cycles (Figure 10).

## 4. Discussion

### 4.1. Anatomical and Histological Basis for AFC-SEG Compliance

**Figure 11:**
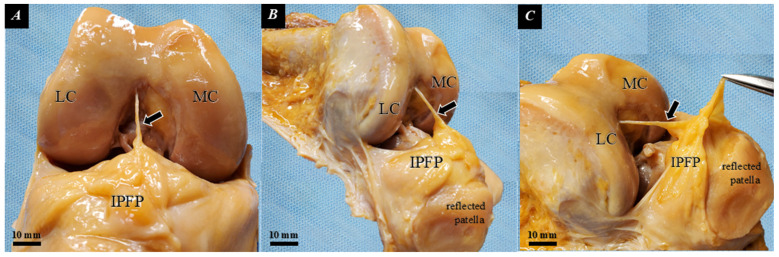
Gross anatomy of the IPP in a Thiel-embalmed specimen, shown at 90° of knee flexion. The IPFP synovium is held with tweezers. (**A**) anterior view; (**B**,**C**) lateral view; MC: medial condyle; LC: lateral condyle; black arrow: IPP.

Our macroscopic and histological analyses were conducted primarily to establish the structural basis for selecting the AFC-SEG as the optimal ultrasonographic target. While the IPFP is a continuous structure, our macroscopic dissection confirmed that it comprises segments with varying degrees of deformability. For instance, gross anatomical observation clearly demonstrated the physical continuity between the IPFP and the IPP, which corresponds to the IPP-SEG (Figure 11). The IPP represents a dense connective tissue structure that widens as it descends anteriorly and inferiorly [18]. Crucially, our findings highlight that a horizontal cleft physically separates this rigid IPP-SEG from the adjacent AFC-SEG [19].

**Figure 12:**
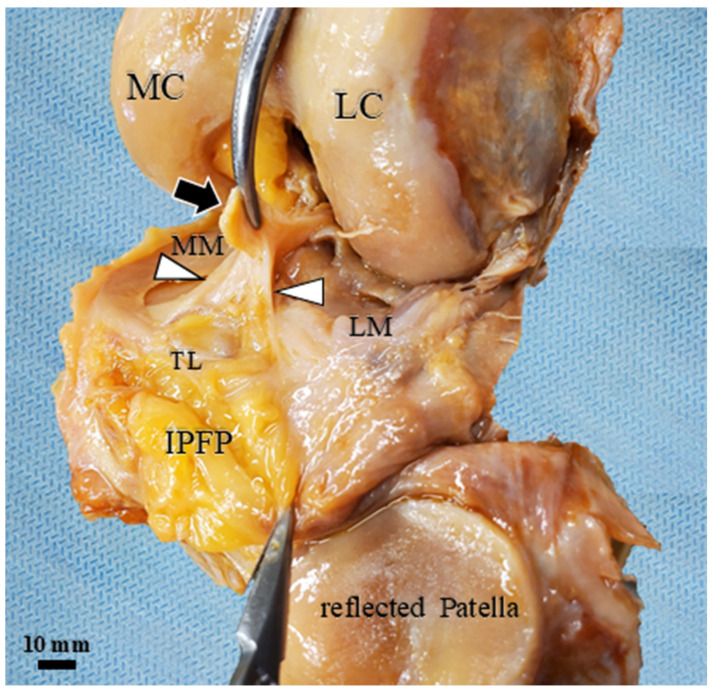
Gross anatomy of the IPFP in a Thiel-embalmed specimen at 130° knee flexion (anterosuperior view). The IPP is completely transected. The synovium covering the ACL (white arrowheads) connects to the tibial plateau under the medial meniscus medially, and to the synovium of the lateral meniscus and the IPFP laterally. MC: medial condyle; LC: lateral condyle; MM: medial meniscus; LM: lateral meniscus; TL: transverse ligament; black arrow: ruptured ACL; white arrowhead: synovial membrane covering ACL.

**Figure 13:**
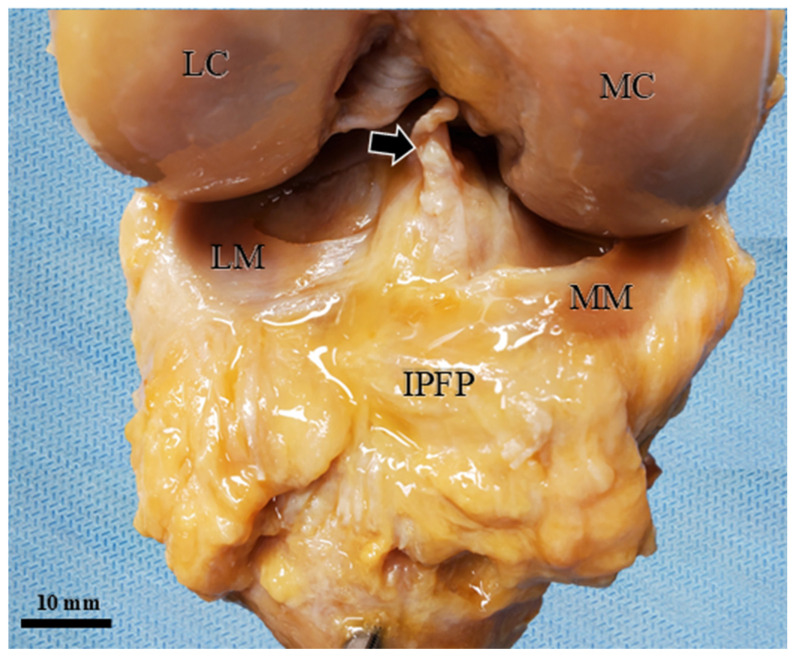
Gross anatomy of the IPFP in a Thiel-embalmed specimen. Anterosuperior view at 130 knee flexion. The synovium extending from the IPFP covers the meniscal surface. The ACL is ruptured (black arrow).

Furthermore, anterior dissection performed after all dynamic measurements provided important anatomical validations. It demonstrated the extensive synovial continuity connecting the IPFP and menisci to the synovium covering the ACL (Figure 12). Importantly, this post-measurement dissection also served as macroscopic evidence confirming that our surgical posterior approach had successfully and completely transected the ACL without disrupting the anterior compartment mechanics (Figure 13).

Combined with its unique follicle-like histological architecture, the structural cleft mechanically isolates the AFC-SEG from denser adjacent tissues, granting it exceptionally high compliance. We posit that this distinct structural deformability justifies why the AFC-SEG is uniquely susceptible to intra-articular pressure changes, making it an ideal and highly responsive surrogate marker for evaluating dynamic joint kinematics.

### 4.2. Biomechanical Mechanisms

**Figure 14:**
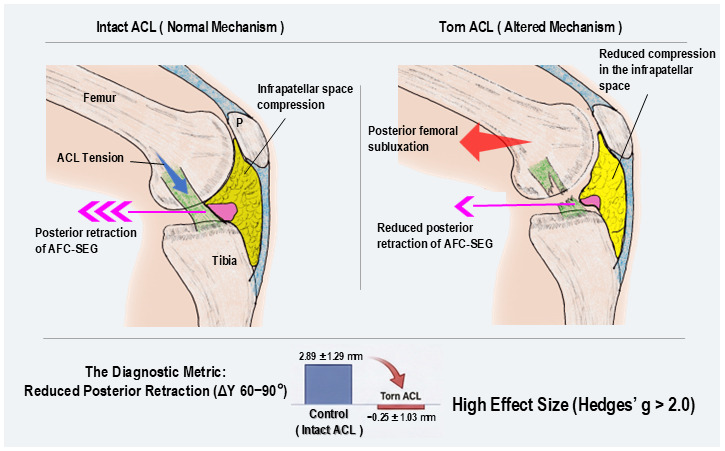
Schematic illustration of the mechanism linking ACL tension to infrapatellar space dynamics. (**Left**) In the intact ACL knee (Normal Mechanism), ACL tension induces posterior retraction of the AFC-SEG (pink region), resulting in compression of the infrapatellar space. (**Right**) In the torn ACL knee (Altered Mechanism), loss of ACL tension leads to posterior femoral subluxation and reduced posterior retraction of the AFC-SEG. (**Bottom**) The bar graph compares the diagnostic metric (Reduced Posterior Retraction, ΔY 60–90°) between the Control and Torn ACL groups. The Torn ACL group shows significantly reduced retraction compared to the Control group, demonstrating a high effect size (Hedges’ g > 2.0). P: patella.

The distinct kinematic alteration directly observed in our study—specifically, the significant loss of posterior AFC-SEG excursion at 90° in the Torn ACL group—provides ex vivo evidence of altered soft-tissue dynamics. We infer that this phenomenon can be mechanistically explained by the interplay between joint compression and soft-tissue compliance. Based on broader biomechanical principles, the intact ACL resists posterior translation of the femur relative to the tibia during flexion [20]. We hypothesize that in ACL-deficient knees, the resultant posterior subluxation of the femur distorts the compressive vectors acting on the IPFP. Under normal physiological compression, the highly compliant AFC-SEG is forced to displace posteriorly. However, when the ACL is torn, this altered skeletal kinematics—potentially combined with the disruption of synovial continuity (via the ligamentum mucosum)—disturbs the normal guidance mechanism, leaving the AFC-SEG abnormally anterior (Figure 14).

It is important to contextualize our marker within existing dynamic ultrasound signs, such as the bony“femoral–tibial step-off” or dynamic anterior tibial translation [9,11]. While these existing metrics quantify global joint instability (skeletal translation), our AFC-SEG tracking reflects intra-articular soft-tissue kinematics [21,22]. Therefore, this method should be viewed not as a replacement, but as a complementary diagnostic tool. It offers unique insight into internal derangement and impingement risks that purely osseous measurements might overlook.

### 4.3. Exploratory Clinical Workflow for ACL Screening

**Figure 15:**
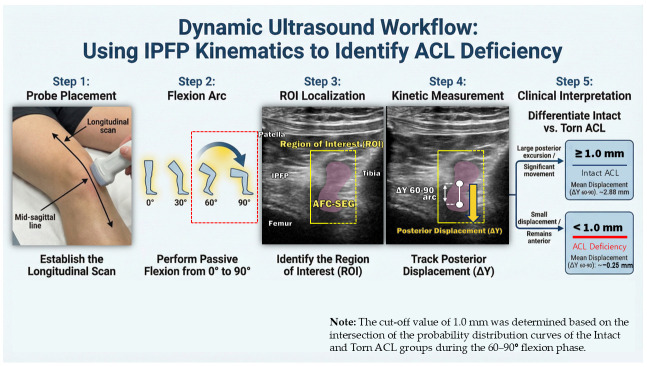
Proposed clinical workflow for non-invasive ACL screening using IPFP kinematics. The protocol utilizes the dynamic behavior of the AFC-SEG to identify ACL deficiency. Steps 1–2: The ultrasound probe is placed longitudinally, and the knee is passively flexed from 0° to 90°(the red frame in Step 2 highlights the 60–90° range as the critical phase for evaluation). Steps 3–4: The Region of Interest (ROI) is focused on the AFC-SEG movement relative to the tibia. The ΔY 60–90° is quantified specifically. Step 5: Clinical interpretation based on the derived cut-off value. A displacement of ≥1.0 mm indicates normal posterior translation (Intact ACL), while a displacement of <1.0 mm indicates abnormal posterior retention (Torn ACL).

To facilitate the clinical translation of our biomechanical findings, we propose an exploratory dynamic ultrasound workflow for ACL screening (Figure 15). This framework focuses specifically on the 60–90° flexion arc, where the kinematic divergence was most pronounced. Based on our preliminary ex vivo statistical analysis, a vertical displacement (ΔY) threshold of 1.0 mm serves as an initial proof-of-concept cut-off value. In this proposed workflow, posterior excursion of the AFC-SEG less than 1.0 mm (or anterior retention) during deep flexion suggests a high probability of ACL insufficiency. This non-invasive assessment could serve as a valuable adjunct to physical examination in acute settings where pain limits manual testing. However, it is crucial to emphasize that this 1.0 mm threshold is strictly exploratory. Future large-scale clinical studies with ROC-based validation in living subjects are required to establish absolute diagnostic criteria.

### 4.4. Limitations

This study has several limitations that must be acknowledged. First, the inclusion of bilateral knees from the same donors introduces potential pseudoreplication. Although we rigorously adjusted for this using a Linear Mixed-Effects Model, future studies with fully independent samples are desirable. Second, the passive flexion protocol utilized in this cadaveric study fundamentally lacks active muscle tone and physiological weight-bearing loads. Because the quadriceps mechanism continuously modulates IPFP dynamics, in vivo validation under active movement is warranted. Third, our specimens were elderly Thiel-embalmed cadavers. While Thiel embalming preserves near-physiological flexibility, these specimens lack active perfusion, and post-mortem alterations in tissue hydration or elasticity are inevitable. Furthermore, adipose tissues in the elderly may exhibit age-related fibrosis compared to those of young athletes. However, the fact that a distinct signal was detectable even in these potentially stiffer tissues suggests the kinematic alteration would likely be even more pronounced in the highly compliant tissues of young patients. Fourth, dynamic ultrasonography is inherently operator dependent. While our study demonstrated excellent intra-rater reliability under controlled-laboratory conditions, inter-rater variability must be rigorously evaluated in future clinical settings. Finally, the insertion of a metallic clip via a 14G needle creates a marker tract that might induce local tissue drag. However, this acts as a systematic, non-differential bias across all groups, meaning it does not confound the relative differences observed.

### 4.5. Future Directions (Translational Plan)

To translate these fundamental biomechanical findings into daily clinical practice, we have formulated a pre-registered translational research plan. We intend to conduct a prospective, blinded in vivo diagnostic accuracy study comprising approximately 60 to 100 knees. In this future clinical trial, our dynamic ultrasonographic assessment will be benchmarked against MRI and/or arthroscopy as the reference standard. We will apply the kinematic metrics established in the current cadaveric models—specifically, the absolute displacement (*y*_2_ − *y*_1_) at 90° and the interval displacement (ΔY 60–90°)—with prespecified diagnostic thresholds. The clinical utility will be rigorously evaluated by reporting the area under the receiver operating characteristic curve (AUC), sensitivity, specificity, and likelihood ratios. Beyond initial diagnosis, future insights suggest this dynamic ultrasonographic tracking could be integrated into acute trauma settings where MRI is inaccessible or time-consuming. Furthermore, it holds potential utility for the longitudinal monitoring of joint kinematics during conservative rehabilitation or post-operative recovery. Exploring the relationship between AFC-SEG kinematics and dynamic weight-bearing activities in future studies will provide a more comprehensive understanding of intra-articular biomechanics.

## 5. Conclusions

In conclusion, this cadaveric study demonstrates that ACL deficiency induces a distinct kinematic alteration in the AFC-SEG of the IPFP, characterized by a significant loss of posterior excursion, resulting in an abnormal anterior retention during deep flexion. However, given the small sample size and ex vivo nature, we prudently avoid making firm diagnostic claims at this stage. Rather, these findings should be interpreted as a foundational proof-of-concept. This dynamic ultrasonographic tracking shows strong potential as a complementary indicator for ACL tears, establishing a robust biomechanical rationale that warrants rigorous validation in future large-scale, in vivo clinical trials.

## Figures and Tables

**Table 1:** Macroscopic and histological characteristics of the five distinct segments of the IPFP.

Segment Abbreviation	Full Name	Macroscopic Anatomical Location	Histological and Structural Features
SPP-SEG	Superficial Proximal Segment	Connects the patella directly to the superficial layer of the patellar tendon.	Densely packed adipocytes. Separated from the SPD-SEG by connective tissue septa.
SPD-SEG	Superficial Distal Segment	Bounded by the patellar tendon, deep infrapatellar bursa, and tibia.	Adipocytes present. Linked to the tibia via the complex connective tissue of the joint capsule.
IPP-SEG	Infrapatellar Plica Segment	Regarded as the extended tissue of the infrapatellar plica (ligamentum mucosum).	High ratio of dense connective tissue mixed with adipocytes. Structurally less deformable.
DSV-SEG	Deep Synovial Segment	Deepest portion near the joint cavity. Separated from the patella by a horizontal cleft.	Characterized by a synovial cell lining on its articular surface.
AFC-SEG **[Target Region]**	Adipo-Follicular Segment	Bounded by SPD-SEG, IPP-SEG, and tibia. Connected to the anterior horns of the menisci.	Contains unique follicle-like spaces. Highly compliant and susceptible to joint pressure changes.

## Data Availability

The data presented in this study are available on request from the corresponding author. The data are not publicly available due to privacy or ethical restrictions.

## Supplementary Materials

The following supporting information can be downloaded at https://www.mdpi.com/article/10.3390/diagnostics16071097/s1, Table S1: Coordinate data of the metallic clip within the IPFP (AFC-SEG) at each recorded angle using ImageJ software on ultrasonic images; Table S2: Differences between the inserted metallic clip position (*x*_2_, *y*_2_) and the anchoring point (*x*_1_, *y*_1_) in each flexion interval.

## Abbreviations

The following abbreviations are used in this manuscript:

ACL	Anterior Cruciate Ligament
AFC-SEG	Adipo-Follicular Segment
ANOVA	Analysis of Variance
AUC	Area Under the Receiver Operating Characteristic Curve
CIs	Confidence Intervals
DSV-SEG	Deep Synovial Segment
H&E	Hematoxylin and Eosin
ICC	Intraclass Correlation Coefficient
IPFP	Infrapatellar Fat Pad
IPP	Infrapatellar Plica
LMM	Linear Mixed-Effects Model
mm	Millimeters
MRI	Magnetic Resonance Imaging
ROI	Region of Interest
SEG	Segment
SPD-SEG	Superficial Distal Segment
SPP-SEG	Superficial Proximal Segment
Δ	Interval Displacement
