# Supplementary material for: Dynamic Ultrasound of the Infrapatellar Fat Pad for Detecting Anterior Cruciate Ligament Deficiency: A Biomechanical Cadaveric Proof-of-Concept Study
Source: Diagnostics (Basel). 2026 Apr 5;16(7):1097. doi: 10.3390/diagnostics16071097 (PMC13072874; doi:10.3390/diagnostics16071097)
Supplement: Supplementary file 1 [file diagnostics-16-01097-s001.zip › diagnostics-4212076-supplementary.pdf]

Table S1. Coordinate data of the metallic clip within the IPFP (AFC-SEG) at each recorded angle using ImageJ software on ultrasonic images

	**Control**								**Sham**								**Torn ACL**							
DNR	1(R)	1(L)	2(L)	3(R)	3(L)	4(R)	4(L)	5(R)	1(R)	1(L)	2(L)	3(R)	3(L)	4(R)	4(L)	5(R)	1(R)	1(L)	2(L)	3(R)	3(L)	4(R)	4(L)	5(R)
**knee angle 0° (mm)**																								
x_1	24.06	18.78	23.05	20.37	21.63	20.12	22.89	19.79	24.65	20.20	16.26	18.53	20.20	18.70	22.47	20.29	23.56	16.93	17.44	17.77	15.68	20.37	20.54	20.29
y_1	11.32	11.07	10.73	10.81	11.23	11.40	11.40	13.25	10.65	10.98	9.72	10.65	9.89	10.98	11.65	11.74	13.25	11.99	10.56	10.56	9.89	11.23	11.49	11.90
x_2	40.07	35.55	38.40	35.21	35.46	38.73	41.16	39.74	39.49	35.63	32.19	35.63	36.64	41.25	41.41	41.50	40.33	34.46	33.37	35.13	32.95	42.92	43.09	39.49
y_2	20.20	21.71	22.72	22.30	23.05	20.12	19.70	22.55	19.62	21.04	22.05	21.71	18.28	18.70	19.95	22.97	23.05	21.97	22.22	24.73	20.71	20.79	19.79	24.40
ΔX	16.01	16.77	15.34	14.84	13.83	18.61	18.28	19.95	14.84	15.43	15.93	17.10	16.43	22.55	18.95	21.21	16.77	17.52	15.93	17.35	17.27	22.55	22.55	19.20
ΔY	8.89	10.65	11.99	11.49	11.82	8.72	8.30	9.31	8.97	10.06	12.32	11.07	8.38	7.71	8.30	11.23	9.81	9.98	11.65	14.17	10.81	9.56	8.30	12.49
**knee angle 30° (mm)**																								
x_1	22.05	17.35	22.47	24.82	19.79	22.30	17.94	20.88	20.12	24.06	18.28	25.32	23.47	21.71	19.95	18.28	15.85	25.82	18.61	19.11	21.71	20.88	19.87	20.12
y_1	10.73	10.98	10.48	10.40	10.48	11.49	11.49	12.74	10.81	9.89	9.89	10.48	10.14	10.98	11.49	11.82	11.82	11.15	10.73	10.73	12.41	11.32	11.82	11.15
x_2	38.31	35.46	39.74	42.76	36.72	42.76	37.22	42.00	36.97	41.41	33.79	43.43	39.32	37.81	42.76	40.07	33.87	42.17	33.12	38.23	36.47	42.00	39.32	42.17
y_2	20.20	21.38	23.56	22.05	21.80	22.55	21.38	24.31	22.38	18.36	21.38	21.88	22.38	23.05	20.29	21.97	23.31	22.30	22.13	25.23	24.65	22.30	18.95	23.22
ΔX	16.26	18.11	17.27	17.94	16.93	20.46	19.28	21.13	16.85	17.35	15.51	18.11	15.85	16.10	22.80	21.80	18.02	16.35	14.50	19.11	14.76	21.13	19.45	22.05
ΔY	9.47	10.40	13.08	11.65	11.32	11.07	9.89	11.57	11.57	8.47	11.49	11.40	12.24	12.07	8.80	10.14	11.49	11.15	11.40	14.50	12.24	10.98	7.13	12.07
**knee angle 60° (mm)**																								
x_1	21.38	18.61	20.37	24.56	15.59	19.70	23.22	20.96	23.39	19.79	16.60	22.64	22.55	22.97	21.21	20.71	15.93	19.03	18.95	19.79	19.95	23.64	26.66	17.94
y_1	11.32	10.48	10.31	10.48	10.40	11.57	11.40	12.49	11.57	10.98	9.47	10.23	9.64	11.74	11.49	11.65	12.41	11.74	10.65	10.65	12.91	10.06	11.99	12.41
x_2	40.16	37.89	40.07	42.00	30.52	38.23	39.74	45.86	41.16	38.06	35.46	37.14	37.81	35.71	41.08	44.10	33.62	34.96	40.16	38.23	35.88	38.06	42.42	38.90
y_2	21.80	23.31	24.06	22.89	24.31	24.82	23.47	25.23	22.22	22.72	23.14	23.39	22.89	24.06	24.14	25.40	23.64	24.40	22.64	25.91	26.16	20.96	21.21	23.98
ΔX	18.78	19.28	19.70	17.44	14.92	18.53	16.52	24.90	17.77	18.28	18.86	14.50	15.26	12.74	19.87	23.39	17.69	15.93	21.21	18.44	15.93	14.42	15.76	20.96
ΔY	10.48	12.83	13.75	12.41	13.92	13.25	12.07	12.74	10.65	11.74	13.67	13.16	13.25	12.32	12.66	13.75	11.23	12.66	11.99	15.26	13.25	10.90	9.22	11.57
**knee angle 90° (mm)**																								
x_1	23.81	23.64	20.04	24.31	16.10	19.70	19.45	22.05	23.39	19.87	17.94	25.49	21.21	19.95	21.88	19.87	15.51	21.55	19.62	21.21	24.73	18.95	24.31	18.70
y_1	11.23	11.32	10.40	10.14	10.73	12.74	12.41	13.83	10.65	10.81	9.98	10.48	9.47	11.32	11.15	11.57	12.07	11.57	9.56	10.23	13.08	10.81	11.57	11.65
x_2	41.08	41.33	37.98	42.84	29.01	34.79	37.31	40.07	40.91	37.56	38.48	42.92	33.20	34.54	39.91	36.22	34.04	36.72	41.08	39.65	37.22	39.49	41.16	38.73
y_2	24.40	26.32	26.58	27.50	25.32	28.84	28.67	29.68	23.22	24.06	26.83	27.33	25.49	27.41	30.68	29.34	22.30	23.47	22.64	25.65	25.99	19.79	20.46	24.31
ΔX	17.27	17.69	17.94	18.53	12.91	15.09	17.86	18.02	17.52	17.69	20.54	17.44	11.99	14.59	18.02	16.35	18.53	15.17	21.46	18.44	12.49	20.54	16.85	20.04
ΔY	13.16	15.01	16.18	17.35	14.59	16.10	16.26	15.85	12.58	13.25	16.85	16.85	16.01	16.10	19.53	17.77	10.23	11.90	13.08	15.43	12.91	8.97	8.89	12.66

DNR = Donor, L = Left knee, R = Right knee, $\Delta X = x_2 - x_1$, $\Delta Y = y_2 - y_1$

Table S2. Differences between the inserted metallic clip position (x_2, y_2) and the anchoring point (x_1, y_1) in each flexion interval

	**Control**								**Sham**								**Torn ACL**							
DNR	1(R)	1(L)	2(L)	3(R)	3(L)	4(R)	4(L)	5(R)	1(R)	1(L)	2(L)	3(R)	3(L)	4(R)	4(L)	5(R)	1(R)	1(L)	2(L)	3(R)	3(L)	4(R)	4(L)	5(R)
**knee angle from 0° to 30° (mm)**																								
ΔX 0-30	0.25	1.34	1.93	3.10	3.10	1.84	1.01	1.17	2.01	1.93	-0.42	1.01	-0.59	-6.46	3.86	0.59	1.26	-1.17	-1.43	1.76	-2.52	-1.43	-3.10	2.85
ΔY 0-30	0.59	-0.25	1.09	0.17	-0.50	2.35	1.59	2.26	2.60	-1.59	-0.84	0.34	3.86	4.36	0.50	-1.09	1.68	1.17	-0.25	0.34	1.43	1.43	-1.17	-0.42
**knee angle from 30° to 60° (mm)**																								
ΔX 30-60	2.52	1.17	2.43	-0.50	-2.01	-1.93	-2.77	3.77	0.92	0.92	3.35	-3.60	-0.59	-3.35	-2.93	1.59	-0.34	-0.42	6.71	-0.67	1.17	-6.71	-3.69	-1.09
ΔY 30-60	1.01	2.43	0.67	0.75	2.60	2.18	2.18	1.17	-0.92	3.27	2.18	1.76	1.01	0.25	3.86	3.60	-0.25	1.51	0.59	0.75	1.01	-0.08	2.10	-0.50
**knee angle from 60° to 90° (mm)**																								
ΔX 60-90	-1.51	-1.59	-1.76	1.09	-2.01	-3.44	1.34	-6.87	-0.25	-0.59	1.68	2.93	-3.27	1.84	-1.84	-7.04	0.84	-0.75	0.25	0.00	-3.44	6.12	1.09	-0.92
ΔY 60-90	2.68	2.18	2.43	4.95	0.67	2.85	4.19	3.10	1.93	1.51	3.19	3.69	2.77	3.77	6.87	4.02	-1.01	-0.75	1.09	0.17	-0.34	-1.93	-0.34	1.09

DNR = Donor, L = Left knee, R = Right knee, $\Delta X = x_2 - x_1$, $\Delta Y = y_2 - y_1$
